# Acute Rheumatic Fever and Kawasaki Disease Occurring in a Single Patient

**DOI:** 10.3389/fped.2020.00562

**Published:** 2020-09-03

**Authors:** Kazuki Iio, Naoya Fukushima, Keiji Akamine, Kazuhiro Uda, Hiroshi Hataya, Masaru Miura

**Affiliations:** ^1^Department of General Pediatrics, Tokyo Metropolitan Children's Medical Center, Tokyo, Japan; ^2^Department of Cardiology, Tokyo Metropolitan Children's Medical Center, Tokyo, Japan; ^3^Department of Nephrology and Rheumatology, Tokyo Metropolitan Children's Medical Center, Tokyo, Japan; ^4^Department of Infectious Diseases, Tokyo Metropolitan Children's Medical Center, Tokyo, Japan

**Keywords:** acute rheumatic fever, diagnostic delay, Kawasaki disease, Kawasaki shock syndrome, rebound phenomenon

## Abstract

Kawasaki disease and acute rheumatic fever are two major causes of acquired heart disease in the pediatric population. Although both conditions are well-known entities, the association between them has never been described. We report herein a case of 6-year-old male patient who first presented with Kawasaki shock syndrome, followed by acute rheumatic fever 1 year later. In contrast to the prompt intervention given for atypical Kawasaki disease, in the present case the diagnosis was significantly delayed during the otherwise typical presentation of acute rheumatic fever. Our case highlights the difficulty experienced by many pediatricians in developed nations in diagnosing acute rheumatic fever due to its comparative rarity. To prevent diagnostic errors, all pediatricians should be alert to the possibility of acute rheumatic fever even if they are practicing in areas where it is not endemic.

## Introduction

Kawasaki disease (KD) and acute rheumatic fever (ARF) are the two leading causes of acquired heart disease in the pediatric populations worldwide.

KD is an acute, self-limiting vasculitis characterized by prolonged fever, mucosal lesions, skin rashes, and cervical lymphadenopathy (1). Since its first description by Tomisaku Kawasaki in 1967, the incidence of KD has increased worldwide, mainly in Asian countries (2). Without treatment, coronary artery aneurysms develop in about 25% of the patients (3).

ARF is caused by an autoimmune reaction to throat infections due to *Streptococcus pyogenes* (4). Its diagnosis is based on a constellation of typical clinical features including carditis, polyarthritis, chorea, erythema marginatum, and subcutaneous nodules (4). It was first recognized as a distinct entity by Thomas Sydenham in 1685 (5). Although its incidence has decreased dramatically in many developed nations, ARF still constitutes a massive burden on several endemic pediatric populations (6). Moreover, 50–70% of ARF patients suffer from rheumatic carditis, which frequently involves mitral and aortic valve regurgitation (4).

Some similarities are known to exist between the pathogenesis of ARF and KD. They are both considered to be triggered by infectious pathogens in genetically pre-disposed individuals (7). Although both conditions are familiar to pediatricians worldwide, the presence of both conditions in a single patient has not been reported thus far. We present herein a case of Kawasaki shock syndrome (KSS) in 6-year-old male patient which was followed by the occurrence of ARF 1 year later.

## Case Presentation

**Figure 1:**
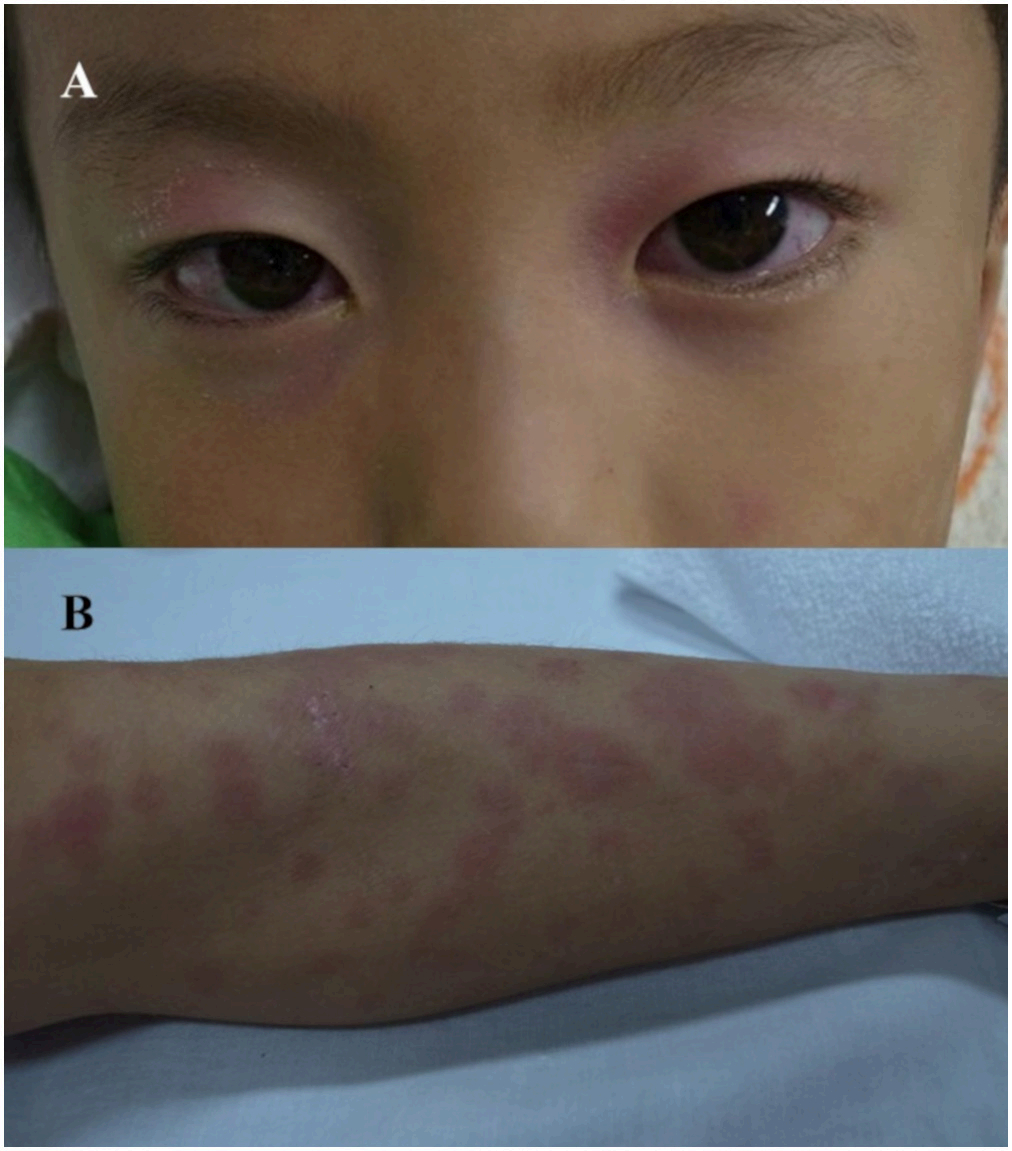
Bulbar injection **(A)**, and macular erythema on the extremities **(B)**.

A 6-year old male patient with history of atopic dermatitis and asthma presented to our hospital with a 4-day history of fever and severe headache. His father and cousin had a history of KD. On presentation, he looked severely lethargic and had tachycardia, cold extremities, and livedo reticularis, all of which improved after normal saline bolus administration. Bulbar injection, cervical lymph node enlargement, and macular erythema on the trunk and extremities were observed. A neck examination revealed prominent nuchal rigidity. A lumbar puncture revealed mild lymphocytic pleocytosis, leading to the tentative diagnosis of meningitis. After admission, antibiotics were administered due to the possibility of bacterial meningitis. However, the fever failed to resolve. A culture and viral polymerase chain reaction assay of blood and cerebral spinal fluid were negative. Due to exacerbation of the macular erythema, cervical lymphadenopathy, redness of the lips, and bulbar injection (Figure 1), KSS complicated with aseptic meningitis was diagnosed on day 6 of illness. Intravenous immunoglobulin therapy 2 g/kg/dose and aspirin 30 mg/kg/day were administered as the initial therapy but proved ineffective. Until the resolution of symptoms, he required another two courses of intravenous immunoglobulin therapy, infliximab administration (5 mg/kg/dose), and methylprednisolone pulse therapy (30 mg/kg/day for 3 days) followed by prednisolone administration (2 mg/kg/day). No coronary artery lesions or valvular lesions were observed throughout the hospitalization. The diameter of the right coronary artery (RCA), left main coronary artery (LMT), and left anterior descending artery (LAD) at the discharge was 2.06 mm (z-score 0.50), 2.55 mm (z-score 0.35), and 1.97 mm (z-score 0.54), respectively.

**Figure 2:**
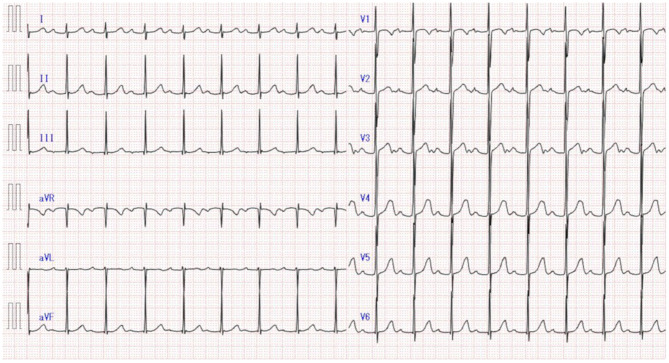
First-degree atrioventricular block detected on electrocardiography at the 1-year follow-up of Kawasaki disease.

One year after discharge, a first-degree atrioventricular block was detected on follow-up electrocardiography (Figure 2) but was no longer visible 2 weeks later on 24-h Holter monitor. A follow-up echocardiography performed at the time was normal, and no other overt symptoms were observed.

Two months later, he presented to the emergency department with arthralgia. He reported first feeling pain in his right knee and ankle, which improved quickly, 1 week before his visit. On the morning of his visit, the pain moved to his left hip and knee. There was no swelling or erythema of the joints. At the follow-up examination 2 days later, the pain had returned to his right knee and bilateral ankles. This time, his right knee joint showed signs of swelling. A blood examination revealed C-reactive protein (CRP) 10.6 mg/dL and erythrocyte sedimentation rate (ESR) 100 mm/h. Acetaminophen was administered, and his joint pain and swelling subsided in 2 days. Based on these findings, viral arthritis was diagnosed, and the follow-up was terminated. No apparent heart murmurs were detected at these visits.

**Figure 3:**
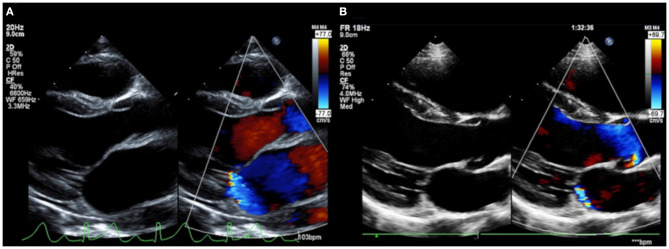
Severe mitral regurgitation on the diagnosis of acute rheumatic fever **(A)**. Mitral regurgitation showed dramatic improvement at the 1-year follow-up **(B)**.

Two months later, he revisited our emergency department with 4-day history of fever, back pain, and right ankle pain. He reported feeling nauseous and lethargic on arrival. Swelling and erythema were prominent in the right ankle. A blood examination revealed CRP 22.4 mg/dL and ESR >100 mm/h. Ferritin was within the normal range at 185.2 μg/L. He was hospitalized and naproxen 20 mg/kg/day was administered, which improved his joint symptoms dramatically. On day 4 of hospitalization, a holosystolic murmur was heard at the apex, and echocardiography revealed severe mitral regurgitation (left ventricular internal diameter in diastole: 44.7 mm; left atrial to aortic root ratio: 1.84; mean pulmonary arterial pressure: 29 mmHg) (Figure 3A). His serum antistreptolysin O (ASO) titer was 926 IU/mL, and a throat culture was positive for M/emm type 6 *Streptococcus pyogenes*. Based on modified Jones criteria, ARF was diagnosed. The diameters of the RCA, LMT, and LAD was 3.10 mm (z-score 2.33), 3.10 mm (z-score 1.64), and 2.70 mm (z-score 1.72), respectively.

A 10-day course of amoxicillin was administered for the *Streptococcus pyogenes* infection, followed by a prophylactic dose of benzathine penicillin G. For the management of carditis, prednisolone 2 mg/kg/day was started. Naproxen was switched to aspirin 30 mg/kg/day. After confirming that the inflammatory markers had normalized at week 3 of hospitalization, the prednisolone dosage was tapered. Prednisolone was administered for 4 weeks in total, and the patient was discharged on day 30 of hospitalization with aspirin alone. However, he returned 3 days after the discharge due to recurrence of arthralgia. CRP, ESR, and ASO were 14.3 mg/dL, 64 mm/h, and 275 IU/mL, respectively. There was no exacerbation of the mitral regurgitation. Because rebound phenomenon was suspected, prednisolone 1 mg/kg/day was initiated on admission, then tapered over 8 weeks. No recurrences of the symptoms were observed thereafter. By the 1-year follow-up, his mitral regurgitation had greatly improved (Figure 3B).

## Discussion

To the best of our knowledge, this is the first case of ARF and KD both occurring in a single patient. Our patient first presented with signs of circulatory failure accompanied by typical KD symptoms, which led to the diagnosis of KSS. His refractory clinical course, characterized by a higher rate of intravenous immunoglobulin therapy resistance, was compatible with KSS (8). His ASO titer at KSS presentation, retrospectively measured by analyzing frozen serum, was negative, indicating that *Streptococcus pyogenes* was not involved in the first febrile episode. In the second episode, the joint symptoms were initially the chief complaint, followed by fever and mitral regurgitation. The present case fulfilled two major and three minor criteria of the modified Jones criteria, and ARF was diagnosed. We consider that his first-degree atrioventricular block was a result of uncomplicated streptococcal infection (9), not a sign of ARF, since he had no joint symptoms, fever, or valvular lesions at that point. *Streptococcus pyogenes* collected from his throat was a pathogenic strain responsible for the ARF (10). The long duration of illness before treatment and steroid use may have predisposed the patient to rebound phenomenon, the reappearance of previously suppressed rheumatic activity upon withdrawal of anti-inflammatory agents (11, 12).

Due to the lack of specific diagnostic tests, the diagnosis of both KD and ARF relies solely on clinical symptoms and biological data (1, 4). Since the major symptoms required for diagnosis are quite different, distinguishing between a “typical” case of KD and ARF is generally not difficult. Mucous symptoms are not seen in ARF while chorea, erythema marginatum, and subcutaneous nodules are absent in KD.

However, unlike the major clinical features, the atypical manifestations of KD are highly diverse (1), and clinical overlaps with ARF can occur in these cases. About 7.5% of KD patients are known to present with arthritis, a major symptom of ARF (13). According to a nationwide survey of KD in Japan, valvular lesions, another major feature of ARF, occurred in 1.5% of KD patients during the acute phase (14). Since *Streptococcus pyogenes* infections concurrent with KD are possible (15), the coexistence of valvular lesions and arthritis with typical KD symptoms may fulfill the diagnostic criteria of both ARF and KD, as actually documented in a case report from Greece (16).

Over the last 50 years, the epidemiologic relationship between KD and ARF had been dramatically changed. ARF continues to be a major cause of morbidity and mortality among children in developing nations and indigenous populations (6). In sharp contrast, KD has replaced ARF to become the most common cause of acquired heart disease among children in developed countries (2, 6, 17). In our hospital, two ARF cases were diagnosed between 2011 and 2018, in contrast to 921 KD cases diagnosed during the same period.

Considering this epidemiologic background, diagnosing the few remaining cases of ARF in developed countries will be challenging, since it is not routinely included in the differential diagnosis. In our case, in comparison with the rapid treatment for KD with atypical presentations, the diagnosis of ARF was delayed despite having a typical clinical course, a reflection of its rarity in Japan. Similar phenomena have been reported in a case series from Canada describing eight cases of ARF which required an average of 88 days to diagnose (18). Since diagnostic delays can subject the patients to further attacks of ARF, which can in turn lead to exacerbation of rheumatic heart disease (19), pediatricians should always be alert to the possibility of ARF even if they are practicing in non-endemic areas. For the timely diagnosis, we need to perform echocardiographic evaluation promptly in patients with unspecified arthritis, which is useful for detecting the subclinical carditis (4).

## Conclusion

We reported the first case of KD and ARF occurred in a single patient. In the present case, the time until the diagnosis of ARF differed greatly from that of KD due to the rarity of the former condition in the developed world. To prevent diagnostic errors, pediatricians of developed countries should always beware of the possibility of ARF when they evaluate the children with typical symptoms of ARF, including joint, skin, or cardiac manifestations.

## Data Availability Statement

All datasets presented in this study are included in the article/supplementary material.

## Ethics Statement

Written informed consent was obtained from the individual(s) and minor(s)' legal guardian/next of kin, for the publication of any potentially identifiable images or data included in this article.

## Author Contributions

KI provided direct care for the patient and drafted the initial manuscript. NF provided direct care for the patient and critically reviewed and revised the manuscript. KA, KU, HH, and MM critically reviewed and revised the manuscript. All authors approved the final manuscript as submitted and agree to be accountable for all aspects of the work.

## Conflict of Interest

The authors declare that the research was conducted in the absence of any commercial or financial relationships that could be construed as a potential conflict of interest.

## Abbreviations

ARF: acute rheumatic fever
ASO: antistreptolysin O
CRP: C-reactive protein
ESR: erythrocyte sedimentation rate
KD: Kawasaki disease
KSS: Kawasaki shock syndrome
LAD: left anterior descending artery
LMT: left main coronary artery
RCA: right coronary artery.
